# Role of non-invasive coronary imaging in stable angina

**DOI:** 10.21542/gcsp.2024.18

**Published:** 2024-03-03

**Authors:** Zahra Adnan, Binay K. Panjiyar, Areeba M. Mehmood, Alekhya Nanisetty

**Affiliations:** 1 Foundation University Medical College, Islamabad, Pakistan; 2 GCSRT PGME Harvard Medical School, Boston, Massachusetts, USA; 3 Sargodha Medical College, Sargodha, Pakistan; 4 Kamineni Academy Of Medical Sciences And Research Centre, Hyderabad, India

## Abstract

Chest pain represents a symptom of significant clinical concern due to the potential for lethal etiologies. Accordingly, it is critical to ascertain the presence of stable angina through various diagnostic tests to inform subsequent therapeutic strategies. Stable angina, while potentially progressing to more severe conditions if left untreated, suffers from a paucity of research regarding its management compared to other more fatal causes of chest pain. Recent advancements in radiological imaging necessitate a re-evaluation of the array and functionality of diagnostic tests, with particular emphasis on prioritizing non-invasive methods such as electrocardiography and echocardiography. This study undertakes a comprehensive review of the literature pertaining to various diagnostic tests for stable angina. We conclude that the management of a patient presenting with chest pain encompasses a continuum of care, beginning with a detailed patient history to estimate pre-test probability and culminating in computed tomography coronary angiography. This continuum is highly individualized, taking into account patient-specific variables, disease burden, and test indications. In an era of rapid research advancement, our findings delineate the optimal sequence of initial diagnostic tests, emphasizing the role of current non-invasive imaging modalities as outlined in standard clinical guidelines.

## Introduction and background

Cardiovascular disease (CVD) represents the primary cause of mortality and morbidity in the United States, in addition to being a significant economic burden, with annual expenditures surpassing 3 trillion dollars and projected to nearly double, reaching 6 trillion dollars by the year 2027^1^. Among the various forms of CVD, coronary heart disease (CHD) accounts for the most substantial portion, necessitating extensive diagnostic procedures, numerous hospital admissions, and a wide array of pharmacological interventions^2^. Annually, an estimated 5% of the U.S. population aged 25 to 64 years are subjected to stress testing for the diagnosis of angina pectoris^3^. Extrapolating this to encompass the entire population of Americans aged 25 and above, which numbers approximately 220 million, it is calculated that in excess of 10 million stress tests are conducted each year within the United States, incurring costs exceeding 11 billion dollars^4^. These figures underscore the imperative necessity for a more efficient, cost-effective strategy for the diagnosis and management of CHD.

We must first define the condition known as stable angina, which serves as a precursory manifestation of myocardial ischemia and is alternatively referred to as angina pectoris or typical angina. This clinical syndrome is distinguished by several features, such as:

• A constricting discomfort or pain located in the substernal region of the chest or radiating to the neck, jaw, shoulder, or arm.
• The onset of symptoms is typically precipitated by physical exertion or emotional stress.
• Symptom alleviation is usually achieved through rest or the administration of nitrates within a timeframe of 5 min^5^.

If all three criteria are met then it is referred to as typical angina, and is atypical if it meets only two. The presence of a single characteristic is indicative of non-anginal or non-cardiac chest pain. Additionally, the specific location and nature of the pain serve as further indicators of its etiology; for instance, pain that is central, squeezing, and gripping in nature is more likely to be ischemic in origin^6^. Stable angina, a chronic condition, is associated with a low but significant incidence of acute coronary events and an elevated mortality rate^7^. Considering the prevalence of angina, affecting approximately one million individuals in the United States, it is crucial to accurately diagnose the condition and stratify risk prior to treatment^5^. Recent shifts in nomenclature have moved towards terms that emphasize the origin of the pain, utilizing descriptors such as ‘cardiac’, ‘possibly cardiac’, or ‘non-cardiac’ rather than ‘typical’ or ‘atypical’ to avoid misconceptions regarding the benign nature of the conditions^8^. Moreover, the initiation of optimized medical therapy (OMT) during the diagnostic process is recommended to mitigate adverse events and alleviate symptoms, unless such interventions are contraindicated^6^.

**Figure 1.**
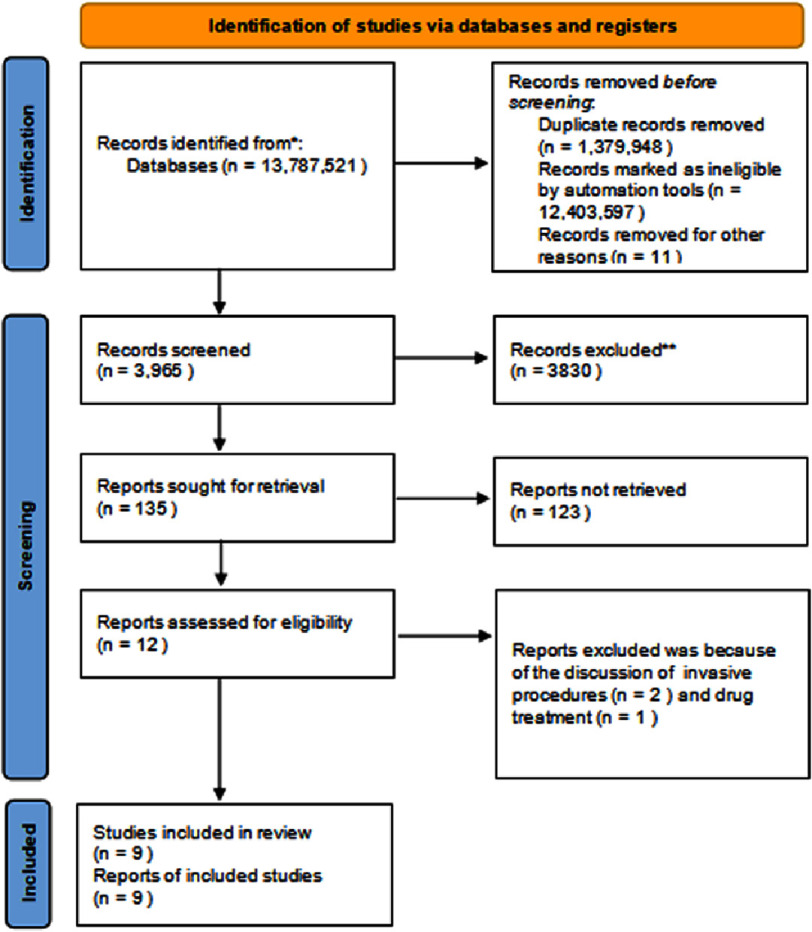
PRISMA flow diagram illustrating the search strategy and study selection process for the systematic review.

The primary objectives in management of stable angina is to reduce or stop symptoms, improve the quality of life and decrease morbidity and mortality. These goals are pursued through a multifaceted approach involving lifestyle alterations, pharmacological intervention, and revascularization via percutaneous or surgical methods^7^. When evaluating the efficacy of an initial invasive strategy against a conservative approach, no significant evidence was found to suggest a reduction in the risk of ischemic cardiovascular events or mortality from any cause over a median follow-up period of 3.2 years^11^. Furthermore, in patients diagnosed with stable coronary artery disease (CAD), revascularization has not demonstrated clear evidence of improved outcomes across major randomized clinical trials, despite extensive research spanning two decades^15^. However, following the outcomes of two pivotal trials, the simultaneous application of functional and anatomical testing has been recommended^16–18^.

Currently, two categories of non-invasive diagnostic tests are available: functional tests, such as exercise electrocardiograms (ECGs) and stress echocardiography (stress Echo), and anatomical tests, including computed tomography coronary angiography (CTCA) amongst others^9^. This raises pertinent questions regarding the indications for these diagnostic tests and the insights they offer.

## Review

### Methods

This review systematically examines clinical research related to non-invasive imaging within the field of cardiovascular medicine. Studies involving animal subjects and those solely addressing the methodological aspects of non-invasive imaging techniques without incorporating clinical data were excluded. Adhering to the Preferred Reporting Items for Systematic Reviews and Meta-Analyses (PRISMA) guidelines for 2020^10^, as depicted in Figure 1, this review employs a structured approach to succinctly encapsulate the article selection process pertinent to the research theme. The methodology relies exclusively on data extracted from peer-reviewed publications, thereby obviating the necessity for ethical clearance. From the initial pool of articles, the step wise process of narrowing the relevant studies is transparently shown at the various stages of the systematic review.

#### Systematic literature search and study selection

We conducted a through search for relevant publications using PubMed (including Medline) and Google Scholar. This search extended to studies referenced within review articles, editorials, and commentaries located on PubMed. An initial list of abstracts was generated and subsequently subjected to independent evaluation for potential inclusion, based on predefined selection criteria centered primarily on stable angina and the utilization of non-invasive imaging modalities. Exclusion criteria were applied to omit review articles and studies involving animal models. The evaluation process was conducted by six reviewers who performed a dual review of each abstract. Any discrepancies arising during the review process were reconciled through discussion.

#### Inclusion and exclusion criteria

We established inclusion and exclusion criteria which is summarized in Table 1.

**Table 1:** Showing the criteria adopted during the literature search process.

	Inclusion Criteria	Exclusion Criteria
(a)	Human studies	Animal studies
(b)	From 2013 to 2023	Studies before 2013
(c)	English text	Non-English texts
(d)	Both genders	Studies discussing imaging
(e)	Age >18 years	Ages below 18 years
(f)	Free papers	Purchasable papers
(g)		Studies not regarding cardiovascular disease

##### Search strategy.

The Population, Intervention/Condition, Control/Comparison, and Outcome (PICO) framework was employed to guide a meticulous review of the literature. This systematic search was executed across electronic databases, including PubMed (encompassing Medline) and Google Scholar Libraries, utilizing a carefully curated list of keywords pertinent to the study objectives, such as “stable angina,” “coronary imaging,” and ”non-invasive imaging.” To enhance the precision and breadth of the search, the Medical Subject Headings (MeSH) terminology was applied specifically to PubMed (inclusive of Medline) and adapted for use within Google Scholar, as detailed in Table 2.

**Table 2:** Showing the search strategy, search engines used, and the number of results displayed.

	Database	Search strategy	Results
(a)	PubMed		12,407,521
		Stable Angina	3,913
		Stable Angina and Non-invasive Imaging	83
(b)	Google Scholar		1,380,000
		Non invasive, Coronary imaging, and Stable angina	52

#### Quality appraisal

To ensure the reliability of our chosen papers, we utilized various quality assessment tools.

For randomized clinical trials included in systematic reviews and meta-analyses, the Preferred Reporting Items for Systematic Reviews and Meta-Analyses (PRISMA) checklist alongside the Cochrane Risk of Bias Tool were employed. The latter is a recommended instrument for evaluating the potential for bias across various dimensions of trial design, execution, and reporting, encompassing aspects such as selection, performance, detection, attrition, and reporting biases.

For the appraisal of non-randomized clinical trials, the Newcastle-Ottawa Scale (NOS) was utilized. This scale is designed to evaluate the quality of non-randomized studies through an assessment across three broad categories: the selection of study groups, the comparability of groups, and the ascertainment of either the exposure or outcome of interest for case-control or cohort studies, respectively.

The evaluation of qualitative research was conducted using the Critical Appraisal Skills Programme (CASP) checklist, as detailed in Table 3. This tool facilitates a systematic assessment of qualitative studies based on rigor, credibility, and relevance.

**Table 3:** Quality appraisal tools used.

Quality appraisal tools used	Type of studies
Cochrane Bias Tool Assessment	Randomized Control Trials
Newcastle-Ottawa Tool	Non-RCT and Observational Studies
PRISMA Checklist	Systematic Reviews
SANRA Checklist	Any Other Without Clear Method Section

**Table 4:** Summary of the results of the selected papers.

Author/Year	Country	Study design	Database used	Conclusion
Ball et al./2018	USA, Italy	Systematic Review	PubMed	FFRCT represents an exciting development in the evaluation of ischemic heart disease. Using advances in imaging and CFD, FFRCT offers a noninvasive diagnostic strategy to identify functionally significant lesions in order to distinguish between patients who can safely avoid ICA and those patients who require revascularization.
Ford et al./ 2020	UK	Systematic Literature Review	Google Scholar	A personalized approach to invasive diagnostic testing permits a diagnosis to be made (or excluded) during the patients’ index presentation
Hoffmann et al./2017	US, Canada	Ransomized Control Trial	PubMed	Coronary CTA, by identifying patients at risk because of nonobstructive CAD, provides better prognostic information than functional testing in contemporary patients who have stable chest pain with a low burden of obstructive CAD, myocardial ischemia, and events.
Morone et al./2022	Italy	Systematic Review	PubMed and Medline	An up-to-date guide for the choice and the interpretation of the currently available noninvasive anatomical and/or functional tests, focusing on emerging techniques, which could provide deeper pathophysiological insights to refine diagnostic and therapeutic pathways in the next future.
Nakano et al./2022	Japan	Systematic Review	Google Scholar	Current guidelines to manage stable coronary artery disease
Pontone et al./2019	Italy, Australia, Sweden	Meta-analysis	PubMed	A negative coronary CT angiography has a higher test performance than other index tests to exclude clinically-important CAD. A positive stress myocardial CT perfusion added to coronary CT angiography, stress cardiac MR, and PET have a higher test performance to identify patients requiring invasive coronary artery evaluation.
Maron et al./2020	USA, UK, Spain, Canada, India, Poland Italy, Germany, Hungary, Japan, France, New Zealand, Russia	Randomized Control Trial	PubMed and Scopus	Among patients with stable coronary disease and moderate or severe ischemia, we did not find evidence that an initial invasive strategy, as compared with an initial conservative strategy, reduced the risk of ischemic cardiovascular events or death from any cause over a median of 3.2 years.
Siontis et al./2018	Switzerland, Canada, UK, Greece	Systematic Review	Google Scholar	For patients with low risk acute coronary syndrome, an initial diagnostic strategy of stress echocardiography or cardiovascular magnetic resonance is associated with fewer referrals for invasive coronary angiography and revascularisation procedures than non-invasive anatomical testing, without apparent impact on the future risk of myocardial infarction.
Rasmussen et al./2019	Denmark, UK	Randomized Control Trial	PubMed and Medline	The results of the Dan-NICAD 2 study are expected to contribute to the improvement of diagnostic strategies for patients suspected of CAD in 3 different steps: risk stratification prior to coronary CTA, diagnostic strategy after coronary CTA, and invasive wireless QFR analysis as an alternative to ICA-FFR.

Furthermore, to mitigate any ambiguity in the appraisal of narrative review articles, the Scale for the Assessment of Narrative Review Articles (SANRA) was implemented. This scale is specifically designed to evaluate the quality of narrative reviews through criteria focused on the transparency and comprehensiveness of the article’s narrative synthesis.

## Results

After searching through three selected databases, PubMed, Medline, and Google Scholar, we extracted 13,787,521 articles. We then carefully reviewed each paper and applied specific criteria, which led to the exclusion of 13,783,556 articles. From the remaining 3,965 papers, we chose not to use 3,830 of them due to duplicates or unsatisfactory titles and abstracts.

We closely examined the remaining 135 papers and excluded 126 more as their content did not meet out inclusion criteria. Finally, we conducted a thorough quality check on the remaining nine papers, which all met our criteria. These nine articles are included in our final systematic review. Table 4 provides a detailed description of each.

## Discussion

### Pretest probability

Prior to diagnostic testing, the first step in patient management is the acquisition of a comprehensive medical history. This enables the clinician to ascertain the pretest probability of disease, which subsequently informs the selection of the most appropriate imaging modality.

For patients presenting with a low adjusted pretest probability of less than 5%—for instance, a young female without coronary risk factors or presenting with atypical chest pain—further diagnostic testing may be deemed unnecessary. Alternatively, such patients might be considered for non-invasive evaluations, such as exercise electrocardiogram (exECG) or assessment of coronary artery calcium (CAC).

Conversely, individuals exhibiting an intermediate to high adjusted pretest probability—for example, an older male with multiple coronary risk factors and abnormal Q waves observed on an ECG—are likely candidates for further non-invasive imaging investigations, barring any indications of acute coronary syndromes. In reality, a significant proportion of patients fall into a pretest probability range of 5–15%, necessitating careful consideration of clinical indicators of CAD, as outlined in Table 5.

**Table 5:** Factors affecting the clinical likelihood of coronary artery disease.

Interview/tests	Suggested components of clinical likelihood
History	Previous cardiovascular disease/polycascular disease
	Comorbidities (e.g., hypertension, dyslipidemia, diabetes, stroke, peripheral artery disease, chronic kidney disease)
	Family history of premature CAD
	Smoking habit
Resting ECG	Abnormal Q waves
	ST-T segment changes
Resting echocardiography	Left ventricular (segmental/diffuse) wall motion abnormality
Blood/urine analysis	Abnormal lipid profile
	Abnormal blood glucose level/tolerance

This approach anticipates a potential escalation in the utilization of imaging modalities, which may, in turn, lead to an increase in false-positive results and associated healthcare costs^6^. It is critical to underscore that the diagnostic accuracy and outcomes derived from non-invasive imaging modalities play a pivotal role in constructing the post-test probability model^12^, thereby facilitating informed clinical decision-making.

**Figure 2.**
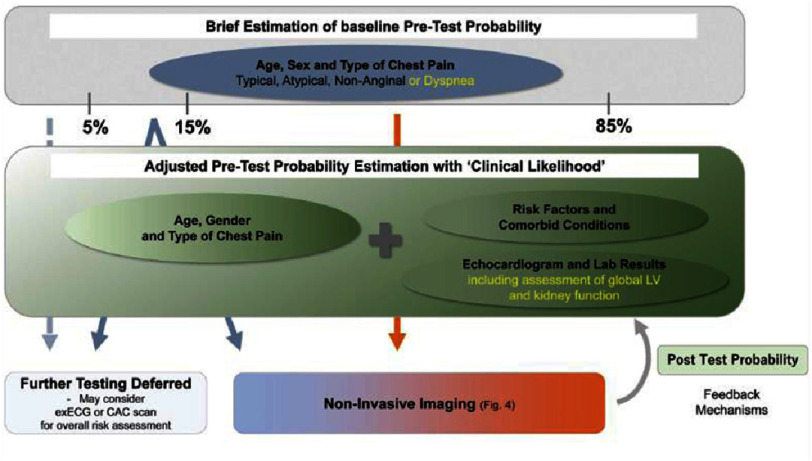
Outline for using pretest probabaility (Reproduced from JSC 2022 guidelines^6^).

If a patient comes with intermediate probability of obstructive CAD, then they are recommended for further workup with non-invasive imaging. In contrast, those with low probability do not require any further investigations as demonstrated in Figure 2. Nevertheless, calculating this probability is underappreciated in contemporary medicine^6^.

**Figure 3.**
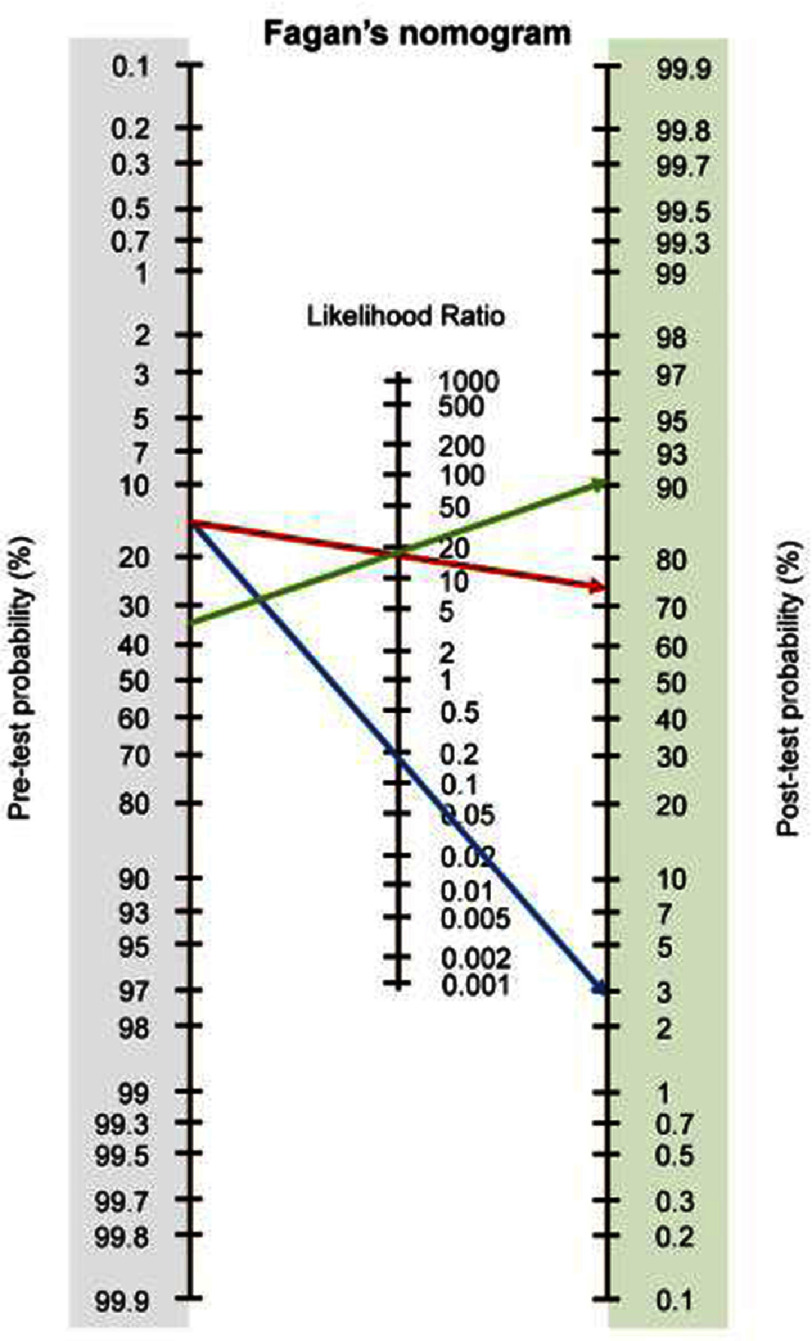
The statistical consequences of predicting pretest probability can be simply described on the Fagan’s nomogram integrating Bayes’ theorem. For example, suppose a patient with 15% baseline pretest probability and a diagnostic imaging test with 85% sensitivity and 95% specificity, the expected post-test probability of having CAD is 75% if the test result is positive (red arrow), whereas it is 3% if the test result is negative (blue arrow). If this patient’s adjusted pretest probability is raised to 35%, the estimated post-test probability is >90% with the positive test result (green arrow). CAD, coronary artery disease. (Reproduced from Watanabe I. 2021^12^).

The results of this estimate can then be used in Fagan’s nomogram integrating Bayes’ theorem^13,14^ shown in Figure 3. Fagan’s nomogram guides physicians to extrapolate from the pretest probability, sensitivity and specificity of the test and the test result to give the post-test probability, which further reinforces or disproves the results^12^.

#### Exercise ECG

The objective of this diagnostic test is to indirectly identify regions of myocardial ischemia through electrocardiographic alterations observed during the exercise and recovery phases. These changes are indicative of a disparity between the myocardial oxygen supply—encompassing both coronary blood flow and the oxygen demand of myocardial work—and the actual oxygen demand during physical exertion. This methodology has been a well-established instrument for evaluating both functional capacity and the chronotropic response to exercise^19^.

Despite JCS guidelines and ACC/AHA 2002 guidelines, the ISCHEMIA trial reported that only 24.5% of participants underwent exercise electrocardiography (exECG)^11^. For patients already diagnosed with CAD, both exECG and functional testing are advocated. However, it is important to note that exECG should not be employed as a diagnostic tool to confirm or rule out stable angina in cases of undiagnosed CAD^7^.

Candidates for exECG are patients:

• Without disabling comorbidity (e.g., frailty, BMI>40kg/m^2^, peripheral artery disease, chronic obstructive pulmonary disease, or orthopedic limitations) and able to perform activities of daily living or 5 metabolic equivalents of exercise
• Without rest ST-T abnormalities (e.g., >0.5-mm ST depression, left ventricular hypertrophy, paced rhythm, left bundle branch block, Wolff-Parkinson-White pattern, or digitalis use)^9^

According to the new ECS guidelines, the usage of exECG is only appropriate when the pretest probability is extremely high (>80%) or low (<19%) due to its limited sensitivity and specificity in diagnosing obstructive CAD. Now, exECG can be performed to confirm the absence of exercised-induced ischemic change in patients with low pretest probability^8^.

**Figure 4.**
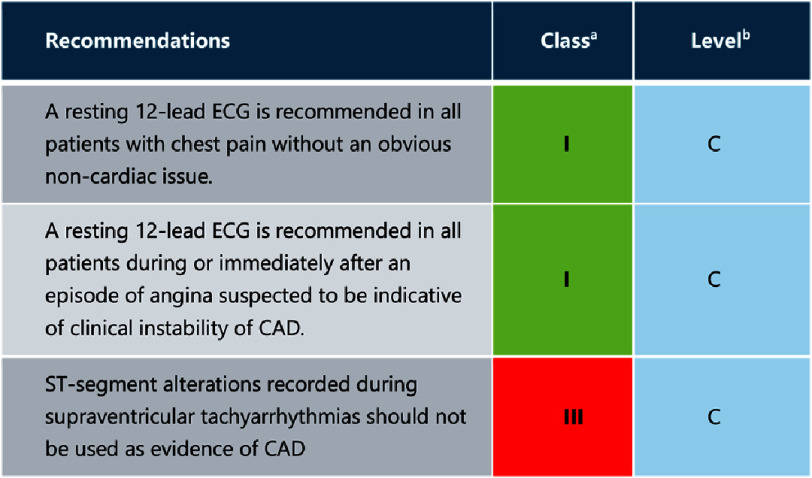
Resting electrocardiogram in the initial diagnostic management of patients with suspected coronary artery disease (Replicated from ESC 2019 Guidelines^8^). CAD = coronary artery disease, CSS = chronic coronary syndrome, ECG = electrocardiogram. a = class of recommendation, b = level of evidence.

#### Stress echocardiography

Echocardiography is deployed to assess myocardial contractility both under resting conditions (Figure 4) and during induced stress, which may be elicited through exercise (utilizing treadmill or bicycle modalities) or pharmacologically (most commonly with dobutamine). It detects the progression of CAD by provoking regional ischemia, which in turn leads to wall motion abnormalities^19^.

Stress trans-thoracic echocardiography (TTE) is particularly good at excluding acute aortic dissection, pericardial effusion, stress cardiomyopathy, and hypertrophic cardiomyopathy, rendering it an invaluable tool for bedside assessment of patients presenting with acute chest pain^9^. Further examination and a deeper comprehension of the incremental benefits afforded by this technology is imperative, with the ultimate goal of identifying the most effective strategies to diminish the morbidity and mortality from CHD^20^.

#### Coronary artery calcium (CAC)

Coronary artery calcium (CAC) scoring can be administered in individuals possessing a low pretest probability to exclude the presence of calcified obstructive CAD. Its widespread availability, cost-effectiveness, and minimal radiation exposure make it a pivotal diagnostic tool. Furthermore, CAC scoring provides significant diagnostic and prognostic insights, even in cases involving extensive calcification of coronary lesions.

A CAC score of zero is commonly interpreted to exclude obstructive CAD, indicative of a favorable prognosis^6^. The application of CAC scoring to distinguish between non-obstructive and obstructive CAD remains a subject of debate, despite growing evidence supporting the role of a zero CAC score in reducing the perceived risk in symptomatic patients with low to intermediate risk profiles.

To date, there have been no extensive studies to establish whether this test should be utilized as a primary diagnostic tool across varying pretest probabilities of CAD^21^. In contrast, stress imaging is recommended as the initial diagnostic approach for individuals with a high pretest probability or a confirmed history of CAD, serving as a crucial component of risk assessment^6^.

#### Computed tomography coronary angiography

Computed tomography coronary angiography (CTCA) enables the direct visualization of coronary arteries through the administration of an intravenous contrast agent, resulting in the generation of an angiogram^6^. Individuals with an intermediate pretest probability of CAD may be considered for CTCA to exclude CAD. However, this modality is contraindicated in patients presenting with severe coronary calcification (for instance, a coronary calcium score exceeding 1,000), uncontrolled or irregular heart rates, or renal dysfunction.

Should obstructive CAD be identified by CTCA, myocardial perfusion imaging (MPI) or fractional flow reserve-computed tomography (FFR-CT, if available and considered suitable) can be applied for further risk stratification. CTCA is the preferred imaging to rule out the presence of CAD^6^ and in the CONFIRM registry, the absence of CAD determined by CTCA was linked to improved prognosis compared to >50% coronary artery stenosis in one or more proximal coronary artery segments^22^.

Jorgensen and colleagues realized that a non-invasive anatomical imaging approach was associated with modifications to treatments, increased downstream invasive testing and subsequent revascularisation, and a lower risk of myocardial infarction (hazard ratio 0.71, 95% confidence interval 0.61 to 0.82) compared with functional testing.

Similarly, a conventional meta analysis of three trials showed a borderline significant reduction of myocardial infarction with CTCA compared to both functional testing and standard care simultaneously (odds ratio 0.69, 95% confidence interval 0.49 to 0.98)^23,24^.

In the SCOT-HEART trial, computed tomography (CT) guided management was associated with better patient outcomes when compared to conventional methodologies. This improvement is attributed, in part, to the more frequent integration of optimized medical therapy (OMT) within the cohort receiving CT-guided management^16,17^.

The ISCHEMIA trial mandated the use of CTCA prior to randomization to exclude patients with left main coronary artery (LMCA) CAD and non-obstructive CAD from the study cohort^11^. However, the positive predictive value for non-LMCA CAD is considered moderate attributed largely to an overestimation of stenosis, especially in moderate to severe coronary calcification^28^. Therefore the introduction of CTCA has not led to a significant decrease in annually performed invasive coronary angiography (ICA) procedures, nor has it significantly increased the number of revascularization procedures following ICA^29^.

Therefore, for the assessment of stable CAD, CTCA has been recommended as the primary imaging modality according to the guidelines issued by the European Society of Cardiology (ESC) and the National Institute for Health and Care Excellence (NICE)^7,8,27^. A negative CTCA outcome is more effective in excluding CAD compared to alternative diagnostic tests, with a success rate of approximately 70–80% among patients. This efficacy is contingent upon the risk profile of the population under examination^25^.

Adding a positive stress myocardial CT perfusion to coronary CTCA, along with stress cardiac magnetic resonance (CMR) and positron emission tomography (PET), enhances the ability to identify patients who may require invasive coronary evaluation^23^. Choosing CTCA as an initial strategy over functional testing does not improve clinical outcomes after a median of 2 years^26^. However, a normal CTCA result is significantly less likely to be associated with major adverse cardiac events (MACE) within the same period, compared to a normal functional test^8^. Contraindications are detailed in Table 6, with the overarching guideline favoring diagnostic accuracy over a comprehensive “triple rule-out” methodology^9^.

**Table 6:**
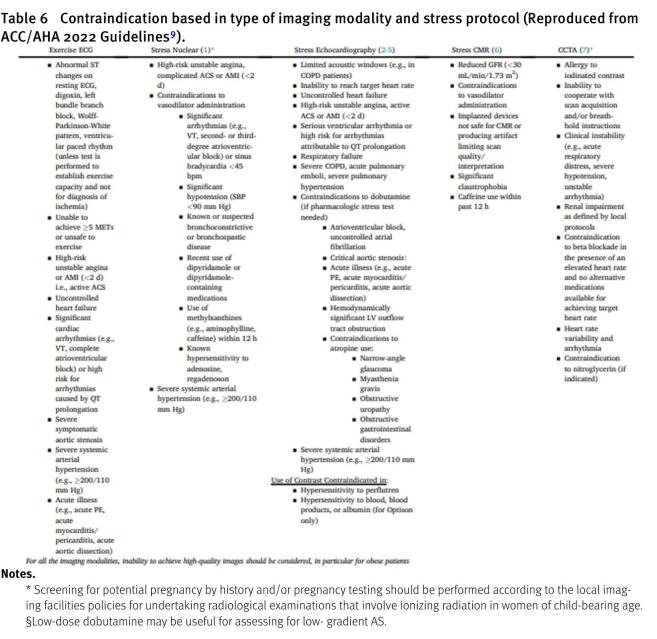
Contraindication based in type of imaging modality and stress protocol (Reproduced from ACC/AHA 2022 Guidelines^9^).

**Notes.**

* Screening for potential pregnancy by history and/or pregnancy testing should be performed according to the local imaging facilities policies for undertaking radiological examinations that involve ionizing radiation in women of child-bearing age. §Low-dose dobutamine may be useful for assessing for low- gradient AS.

In instances of obstructive CAD excluding LMCA involvement, the integration of functional testing prior to proceeding with invasive coronary angiography is typically recommended, particularly for cases of stable CAD where such invasive procedures are deemed not cost-effective despite their similar safety profiles^30^.

The PROMISE trial, in which 13.9% of the intermediate risk group, from more than 4,000 suspected CAD patients, who had obstructive CAD (>50% stenosis in major vessels), showed that >50% of patients previously defined as having an intermediate probability (15–85%) must be reclassified as <15% in the new guidelines^31^.

#### Functional flow reserve

Functional flow reserve (FFR) is the ratio of maximal blood flow through the coronary artery distal to a stenotic lesion, compared to normal blood flow. Traditionally, it is measured in the cardiac catheterization lab using a pressure wire and administering an vasodilator (intracoronary or intravenous) to produce maximal blood flow^17^. As an example, a FFR value of 0.75 means that a stenosis is causing a 25% drop in pressure across the lesion, which means that maximal blood flow is equally reduced by 25%. Recent large trials have demonstrated the viability of FFR as a test to assess the necessity of revascularization, especially for patients with stable CAD. Due to recent advances in imaging, FFRCT offers a noninvasive diagnostic option to identify functionally significant lesions in order to distinguish between patients who can safely avoid ICA versus patients who require revascularization^35^.

The Fractional Flow Reserve Versus Angiography for Multi vessel Evaluation 2 (FAME 2) trial showed a reduction in MI and urgent revascularization in the long term after FFR-guided estimation for performing percutaneous coronary intervention (PCI) (positive if FFR value ≤0.80)^32^.

Based on a high sensitivity for identifying obstructive CAD, increasing evidence supports the use of FFR-CT^33^. In the ISCHEMIA trial, FFR was required in almost 20% of patients undergoing PCI where invasive coronary angiographic findings were different than with non-invasive testing^11^. A multicenter prospective registry revealed that revascularization of lesions exhibiting a FFR greater than 0.8 did not correlate with an enhancement in the one-year event rate, encompassing outcomes such as death, stroke, myocardial infarction (MI), and subsequent revascularization, when compared to a conservative management approach^34^. However, deferred lesions with lower FFR values were associated with a higher incidence of target vessel-related MI and revascularization requirement^23^.

The Myocardial Perfusion Cardiac Magnetic Resonance vs. Angiography and FFR to Guide the Management of Patients with Stable Coronary Artery Disease (MR-INFORM) study demonstrated that cardiac magnetic resonance (CMR)-based stress tests were comparable to FFR in terms of prognostic assessment, moreover, CMR-guided management strategies significantly decreased the need for ICA or revascularization^36^.

FFR-CT is useful to evaluate the functional significance of intermediate stenoses on CTCA, especially in the cases of multivessel disease, to determine the blocked artery^37^. Supplementing FFR-CT to CTCA increases specificity, positive predictive value, and accuracy over just CTCA^38^. The 1-year outcomes from the international multicenter prospective ADVANCE FFRCT Registry analyzing around five thousand patients demonstrated low rates of events in all patients, with less revascularization and a downward trend of major adverse cardiovascular events and significantly lower cardiovascular deaths or myocardial infarction in patients with a negative FFR-CT versus patients with abnormal FFR-CT values^39^. FFR-CT can be used as an alternative functional assessment tool concurrently with other non-invasive functional imaging tests. The use of FFR-CT can assist in avoiding unnecessary invasive coronary angiography^40,41^, however its true cost-effectiveness is still up for debate^42^.

#### Stress Cardiovascular Magnetic Resonance Imaging

Stress CMR represents a sophisticated cross-sectional imaging technique that captures two-dimensional (2-D) or three-dimensional (3-D) images of the heart. During pharmacological stress, a contrast agent is injected and first pass perfusion images can be used to identify areas of ischemia or wall motion abnormalities^9^. This technique offers numerous benefits, including the absence of radiation exposure and superior image resolution^43^, providing significant diagnostic utility in stratified medicine^45^. Furthermore, in a meta-analysis comparing it with Invasive Coronary Angiography-Fractional Flow Reserve (ICA-FFR), Stress CMR demonstrated a sensitivity of 90% and a specificity of 87%^43^. Moreover, for women the sensitivity was 84% and specificity was 78% compared to men who had 89% and 71% respectively^44^.

#### Single Photon Emission CT-Myocardial Perfusion Imaging (SPECT)

In this procedure, an intravenous radioactive tracer for myocardial perfusion is administered to assess cardiac perfusion and function both during exercise/ pharmacological stress and in a resting state. This methodology is utilized to detect myocardial ischemia, infarction, and evaluate ventricular function^9^.

**Table 7:** COR and LOE for Non-Invasive Imaging in Patients with Suspected Stable CAD. CCTA Coronary computed tomography angiography SPECT Single Photon Emission CT- Myocardial Perfusion Imaging CMR cardiac magnetic resonance CAD coronary artery disease PTP pretest probability CL clinical likelihood FFR-CT Functional flow reserve computed tomography OMT optimized medical therapy LMCA left main coronary artery CAC Coronary artery calcium exECG Exercise electrocardiography (Reproduced from ACC/AHA 2022 Guidelines [9])

	COR	LOE
Non-invasive anatomical (CCTA) or functional imaging test (SPECT, stress CMR, or stress echocardiography) is recommended for the diagnosis of CAD and assessment of event risk in patients with intermediate or high PTP of CAD	I	A
It is recommended to choose appropriate non-invasive imaging modality based on the PTP/CL sequence and patients’ characteristics (e.g., heart rate/bundle-branch block/artificial pacemaker, renal dysfunction, drug allergy/exercise intolerance, or risk of radiation exposure)	I	C
Complimentary functional tests (i.e., functional imaging tests and FFR-CT) should be considered for risk assessment or in patients whose findings on CCTA are inconclusive	IIa	B
Invasive coronary angiography should be considered for the diagnosis of CAD prior to titration of OMT when findings on non-invasive imaging tests are suggestive of LMCA or LMCA-equivalent disease, or symptoms deteriorate during diagnostic work-up	IIa	B
CAC scan or exECG may be considered as an optional test to help rule out CAD in asymptomatic or minimally symptomatic patients with low PTP	IIb	B
Invasive coronary angiography should be avoided prior to initiation and titration of OMT unless non-invasive imaging test suggest evidence of LMCA or LMCA-equivalent disease	III (Harm)	B

**Figure 5.**
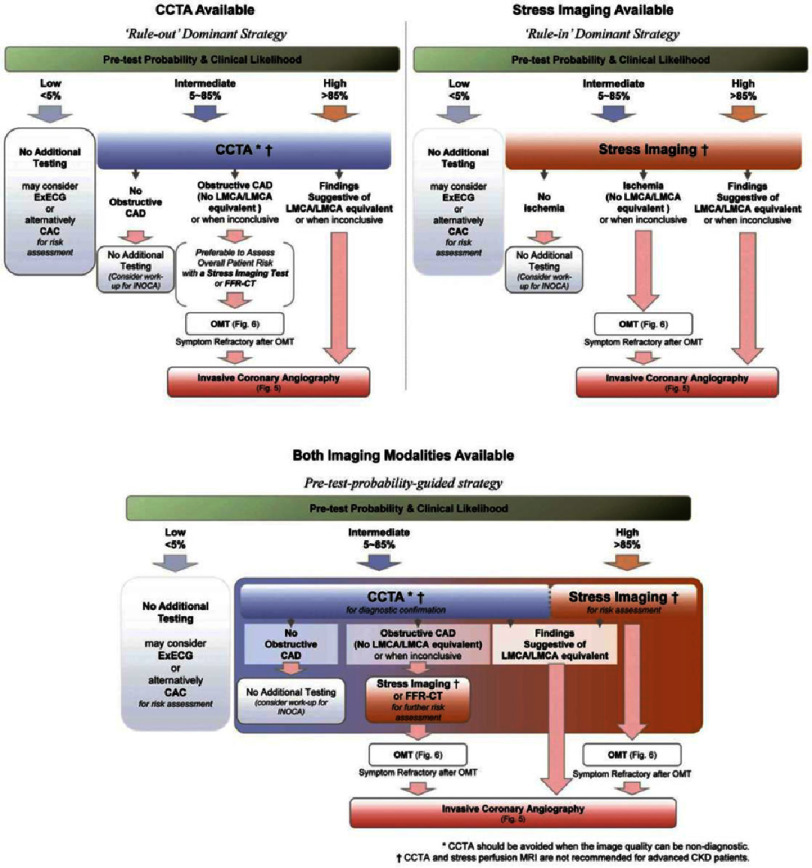
Optimal diagnostic flowchart for non-invasive imaging. The flow of the proposed diagnostic pathway depends on the pretest probability/clinical likelihood sequence and availability of non-invasive testing. If a CT scanner is the only imaging device available, non-obstructive coronary artery should first be ruled out by CCTA (“rule-out strategy”, Left-upper panel). Ideally, FFR-CT analysis or stress imaging techniques are applied before invasive coronary angiography for further risk assessment. If the institution is experienced with functional stress imaging, it is suitable to mainly apply those imaging techniques for diagnosis and risk stratification (“rule-in strategy” Right-upper panel). In institutions capable of performing multi modal imaging (“pretest probability-guided strategy”, Lower panel), CCTA is the preferred imaging to rule out the presence of CAD, whereas stress imaging is preferred as an initial imaging test in patients with a high pretest probability or known history of CAD for risk assessment. CAC, coronary artery calcium; CAD, coronary artery disease; CCTA, coronary computed tomography angiography; exECG, exercise ECG; FFR-CT, fractional flow reserve-computed tomography; INOCA, ischemia with non-obstructive coronary artery disease; LMCA, left main coronary artery; OMT, optimized medical therapy (Replicated from JCS 2022 Guidelines^6^).

### Limitations

Despite our best efforts, our literature review has limitations; we limited our analysis to English articles published within the last 10 years. We also used only free articles, and our study was limited to English papers on stable angina and non-invasive imaging. More research is needed for specific conclusions.

## Conclusion

An initial estimation of pretest probability is critical. For individuals with suspected or confirmed stable CAD presenting with intermediate or high pretest probability, the recommendation is to proceed with non-invasive imaging modalities (Table 7) (Figure 5). Subsequent to this, ICA becomes the preferred course of action if non-invasive tests indicate the possibility of LMCA or LMCA-equivalent disease, as detailed in Table 8. However, the presence of moderate or severe ischemia, in isolation, does not necessitate further invasive interventions. In scenarios where a patient exhibits a low pretest probability (<5%), additional testing might not be required; however, exercise electrocardiogram (exECG) or CAC scan could be considered for further risk stratification^6^.

**Table 8:** High Risk Anatomical (for CACT) and Ischemic (for Functional Imaging) Features Suggestive of LMCA/LMCA-equivalent Disease.

Modality	Findings suggestive of LMCA/LMCA-equivalent disease
CTCA	≥50% obstruction of LMCA
	Significant stenosis in proximal LAD and dominant LCx
	Significant stenosis in proximal LAD and dominant RCA
SPECT	Inducible ischemic area >10% of left ventricular myocardium
	Post-ischemic myocardial stunning/transient ischemic dilatation
Stess CMR	Stress perfusion deficit (e.g., >4/32 LV segments)
	Stress-induced dysfunctional motion (e.g., >3/16 LV segments)
Stress echocardiography	Stress-induced hypokinesia/akinesia (e.g., ≥3/16 LV segments)

Something that is frequently overlooked is the pretest probability, an assessment tool that should be first line and our article its underuse, leading to false positive results or finding benign conditions, which can lead to over treatment. Another point of note is the overuse of exercise ECG in regular practice. Although a useful screening tool, it needs to be used upon the right indications.

We highlight the benefits of a stepwise approach starting from the non-invasive imaging modalities. Standard guidelines have, till now, still not encompassed the newer developments and investigations, and so we do not appreciate outliers from the sphere of modern cardiology, something we must attempt to rectify.

## Additional information

## Disclosures

*Conflict of Interest:* In compliance with the ICMJE uniform disclosure form, all authors declare the following:

*Payment/services info:* All authors have declared that no financial support was received from any organization for the submitted work.

*Financial relationships:* All authors have declared that they have no financial relationships at present or within the previous three years with any organizations that might have an interest in the submitted work.

*Other relationships:* All authors have declared that there are no other relationships or activities that could appear to have influenced the submitted work.

## Authorship

*Conceptualization:* Binay K. Panjiyar, Zahra Adnan

*Software:* Zahra Adnan

*Writing - Original Draft Preparation:* Zahra Adnan

*Writing - Review & Editing:* Zahra Adnan, Binay K. Panjiyar, Areeba M. Mehmood, Alekhya Nanisetty
